# Thyroid hormone regulates adhesion, migration and matrix metalloproteinase 9 activity via αvβ3 integrin in myeloma cells

**DOI:** 10.18632/oncotarget.2205

**Published:** 2014-07-13

**Authors:** Keren Cohen, Nir Flint, Shachar Shalev, Daniel Erez, Tal Baharal, Paul J. Davis, Aleck Hercbergs, Martin Ellis, Osnat Ashur-Fabian

**Affiliations:** ^1^ Translational Hemato-Oncology Laboratory, The Hematology Institute and Blood Bank, Meir Medical Center, Kfar-Saba, Israel; ^2^ Department of Human Molecular Genetics and Biochemistry, Tel Aviv University, Tel Aviv, Israel; ^3^ Sackler Faculty of Medicine, Tel Aviv University, Tel Aviv, Israel; ^4^ Department of Medicine, Albany Medical College, Albany, NY, USA; ^5^ Radiation Oncology, Cleveland Clinic, Cleveland, OH, USA

**Keywords:** Integrin, myeloma, thyroid hormone, MMP-9, adhesion

## Abstract

Thyroid hormone (3,5,3′-triiodothyronine, T3; L-thyroxine, T4) enhances cancer cell proliferation, invasion and angiogenesis via a discrete receptor located near the RGD recognition site on αvβ3 integrin. Tetraiodothyroacetic acid (tetrac) and its nanoparticulate formulation interfere with binding of T3/T4 to the integrin. This integrin is overexpressed in multiple myeloma (MM) and other cancers. MM cells interact with αvβ3 integrin to support growth and invasion. Matrix metalloproteinases (MMPs) are a family of enzymes active in tissue remodeling and cancer. The association between integrins and MMPs secretion and action is well established. In the current study, we examined the effects of thyroid hormone on myeloma cell adhesion, migration and MMP activity.

We show that T3 and T4 increased myeloma adhesion to fibronectin and induced αvβ3 clustering. In addition, the hormones induced MMP-9 expression and activation via αvβ3 and MAPK induction. Bortezomib, a standard myeloma treatment, caused a decrease in activity/quantity of MMPs and thyroid hormone opposed this effect. RGD peptide and tetrac impaired the production of MMP-9 in cell lines and in primary BM cells from myeloma patients.

In conclusion, thyroid hormone-dependent regulation via αvβ3 of myeloma cell adhesion and MMP-9 production may play a role in myeloma migration and progression.

## INTRODUCTION

Multiple myeloma (MM) is a plasma cell neoplasm that primarily affects elderly patients [1]. MM is characterized by the accumulation and localization of malignant plasma cells in the bone marrow (BM), leading to disordered hematopoiesis. Despite significant progress in the management of MM with bortezomib and other novel therapeutic agents, this disease remains highly refractory to therapy[2].

Growing evidence has suggested that the aggressiveness of MM cells and resistance to chemotherapy is partly governed by cellular adhesion between the MM cells and BM stromal cells (BMSCs) as well as extra cellular matrix (ECM) proteins, leading to growth, proliferation and invasion of the malignant clone[2, 3]. The progression and homing of MM cells to the BM is facilitated by the proteolytic degradation of ECM proteins and BM components by a family of matrix metalloproteinases (MMPs) [4]. MMP-9 is one of the most important members of the family and is constitutively active in both human and murine MM cells, contributing to cell growth, invasion, angiogenesis and bone degradation [5-8]. The activation of MMP-9 is not regulated by interleukin-6 (IL-6), the major myeloma cell growth factor, or by other cytokines involved in multiple myeloma [7].

It has recently been shown that integrin-mediated cell interaction with matrix molecules, particularly fibronectin, is the strongest inducer of MMP's production and activation in cells of lymphoid origin[9, 10]. Integrins are a family of cell surface receptors that take part in the interactions between BMSCs, ECM proteins and the MM cells. Among these integrins, αvβ3 plays a pivotal role in MM, engaging in adhesion, invasion and migration [11-13]. We have recently shown that thyroid hormone, via a novel receptor on the αvβ3 integrin[14] that is proximal to the RGD recognition site, acts as growth factors in MM [15]. Tetraiodothyroacetic acid (tetrac) and its nanoparticulate formulation (tetrac-NP) are selective T4/T3 blockers at the αvβ3 integrin site^16,17^. In the current work we were have pursued the effects of the hormones on adhesion, migration and MMP-9 production—and possibly to block these actions—in myeloma cell lines as well as in primary bone marrow cells from myeloma patients. We found that thyroid hormone increase plasma cell adhesion to fibronectin and RGD and to increase cell migration and MMP-9 activation. Moreover, the hormone opposed MMP-9 reduction by bortezomib. These actions are regulated via the αvβ3 integrin and are MAPK-mediated. Disruption of the thyroid-integrin signaling by use of RGD or tetrac, a specific blocker of the thyroid hormone binding site upon the integrin [16, 17], impaired the production of MMP-9 in myeloma cell lines and primary BM cells from myeloma patients.

## RESULTS

### Thyroid hormone increases MM cell adhesion and migration

The RGD sequence is the cell attachment site of a large number of adhesive extracellular matrix proteins, including fibronectin. The interaction of myeloma plasma cells with these proteins is involved in migration of the malignant clone. The regulation of adhesion and migration by thyroid hormone was previously reported in normal physiological processes [18-21] and cancer [22, 23], but has not been implicated in multiple myeloma.

**Figure 1:**
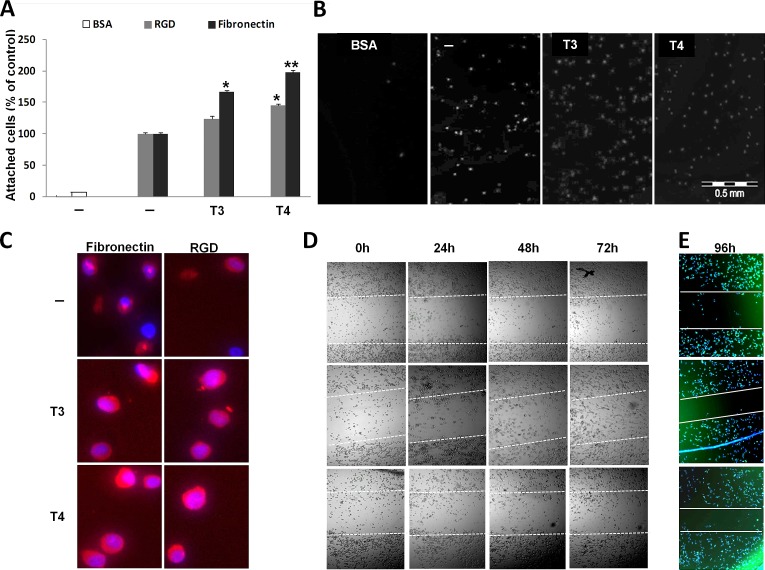
T3 and T4 increase MM cell adhesion and migration CAG cells (100,000/96-well plate) were incubated in serum-free media overnight with T3 (1 nM) or T4 (100 nM) and seeded on BSA/RGD/fibronectin pre-coated plates for 30 min. Cells were (A) counted (% of control), (B) visualized and (C) stained for αvβ3 expression. Scratch wound healing assay in response to T3/T4 for 24-96 h was (D) visualized and (E) stained for cell nucleus (blue) and actin filaments (green) after 96 h. Experiments were repeated twice in triplicates. *p<0.05, **p<0.005.

In order to assess whether T3 and T4 could modulate interactions between myeloma cells and fibronectin/RGD, CAG myeloma cells (100,000/96-well plate) were grown overnight under serum-free conditions and treated with T3 (1 nM) or T4 (100 nM) overnight. The next day, the cells were collected and an equal number of cells were seeded on fibronectin/RGD pre-coated plates for 30 min and the number of adhered cells was assessed. BSA-coated wells served as negative control. Results (Figure 1A) indicate that T3 and T4 increased cell adhesion to RGD by 24% and 44% (p< 0.05), respectively. This effect was further enhanced in fibronectin coated wells by 66% (p< 0.05) following T3 treatment and by 98% (p< 0.005) in T4 treated cells. Representative images from fibronectin-coated wells are depicted in Figure 1B. The cells were further stained by PE-conjugated αvβ3 integrin antibody (LM609) and by a nuclear dye (Hoechst 33342). Representative results (Figure 1C) indicate that T3 and T4 increase αvβ3 abundance on fibronectin- and RGD-adhered cells. Next, to study the effect of thyroid hormones on directional cell migration *in vitro*, a scratch wound healing assay was conducted (Figure 1D). CAG cells (100,000/96-well plate), under serum-free conditions, were plated and a gap in the cell monolayer was created. Fresh T3 (1 nM) or T4 (100 nM) was added every day for 24-96 h. Images were captured by light microscopy equipped with a camera, at the beginning (0 hours) and at regular intervals during cell migration to close the wound (24, 48, 72h). The gap borders throughout the experiment are marked by white dashed lines. Cell migration and growth towards the center of the gap, thereby filling up the gap, was observed in T3 and T4 treated cells and occurred on a time-dependent basis. In untreated cells, a lower migration rate was seen. To further facilitate visualization of closure of the gap borders, cells from the same experiments, after 96h of incubation, were stained for cell nucleus (Hoechst 33342) and actin filaments (Phalloidin) and imaged using a fluorescent microscope. While the gap borders remained almost at their original width in untreated cells, closure of the gap borders following treatments with both hormones was observed (Figure 1E).

### Thyroid hormone induces MMP-9 expression, protein level and activation via αvβ3- MAPK pathway in MM cells

**Figure 2:**
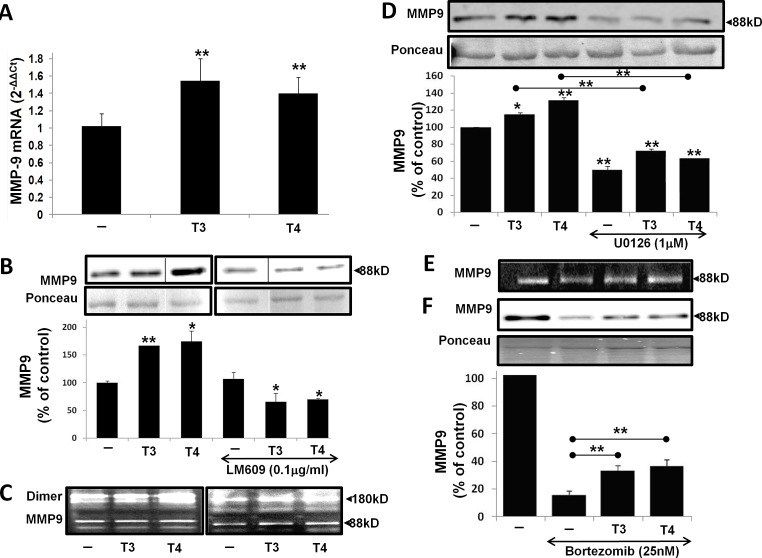
T3/T4 increase MMP-9 mRNA expression, protein level and activation via the MAPK pathway and oppose bortezomib CAG cells (100,000/96-well plate) were incubated in serum-free conditions overnight with T3 (1 nM) or T4 (100 nM) in the presence/absence of U0126 (1 μM), LM609 (0.1 μg/mL) and bortezomib (25 nM) and evaluated for (A) MMP-9 mRNA by real-time PCR. Results were repeated 4 times in duplicate and expressed as fold-change (2^−ΔΔCt^) relative to control cells. (B-F) MMP-9 protein level was measured by western blots and activation by gel zymography. Experiments were repeated at least twice in duplicate. Results are presented as fold of control (average ± SD), *p<0.05, **p<0.005.

Migration of MM cells involves proteolytic degradation of ECM proteins by MMP's, primarily MMP-9. We determined the effect of thyroid hormone on MMP-9 expression, secretion and activation. CAG cells were seeded (100,000/96-well plate) under serum-free conditions for 24 h and treated overnight with T3 (1 nM) or T4 (100 nM) in triplicate. Cells were collected for RNA extraction and results (Figure 2A) show a significant (p< 0.005) increase in MMP-9 mRNA expression by T3 (1.54-fold) and T4 (1.4-fold). Next, MMP-9 protein levels were measured in the conditioned medium of the cells. The pro enzyme MMP-9 (92kD) is cleaved, yielding active enzyme at 88 kD. CAG cells were treated overnight in triplicates with the hormones in the presence/absence of 0.1μg/mL αvβ3 blocking antibody (Clone LM609). Concentrated conditioned medium samples were loaded in two parallel gels and developed together. Results (Figure 2B), from representative lanes (skipping lanes are clearly marked by separating lines), indicate a significant increase in the 88kD active form of MMP-9 in response to T3 (1.66-fold, p<0.005) or T4 (1.74-fold, p<0.05) in comparison to untreated cells. A significant reduction to 0.65 (p<0.005) was observed when the cells were treated with the hormones in the presence of αvβ3 blocking antibody, indicating involvement of the integrin. The next step was to evaluate the effect of T3 (1 nM) or T4 (100 nM) on MMP-9 activation by performing gel zymography in two MM cell lines, CAG (Figure 2C, left panel) and RPMI-8226 (Figure 2C, right panel). An increase in the active form of MMP-9 (88kD) was evident in both cell lines. In CAG, 1.3-fold increase by T3 and 1.51-fold by T4 (p<0.005) was documented and 1.3-fold increase by T3 and a significant 2.3-fold increase by T4 (p<0.005) were observed in RPMI-8226 cells. An increase in the pro-dimer MMP-9 (180kD) was also observed. Comparable results were obtained in the ARK cell line (data not shown). In order to assess whether thyroid hormones-induced MMP-9 is MAPK dependent, CAG cells were treated with the hormones in the presence or absence of the MEK1/2 inhibitor, U0126 (1 μM). Results (Figure 2D) indicate that the induction of active MMP-9 protein by T3 and T4 was significantly reduced (p<0.005) in the presence of the MAPK inhibitor. Similar results were obtained in RPMI-8226 cells (data not shown). Next, we examined whether thyroid hormone may antagonize the effect of bortezomib (25 nM) on MMP-9 level and activation. CAG cells were incubated overnight with bortezomib (25nM) in the presence or absence T3 (1 nM) or T4 (100 nM). Bortezomib reduced MMP-9 activation to 0.6 (Figure 2E) and protein level to 0.15 (Figure 2F) which were significantly antagonized (p<0.005) in the presence of either hormone. Similar results were obtained in RPMI-8226 and ARK cells (data not shown).

### Thyroid hormone activation of MMP-9 is down-regulated by RGD peptide

**Figure 3:**
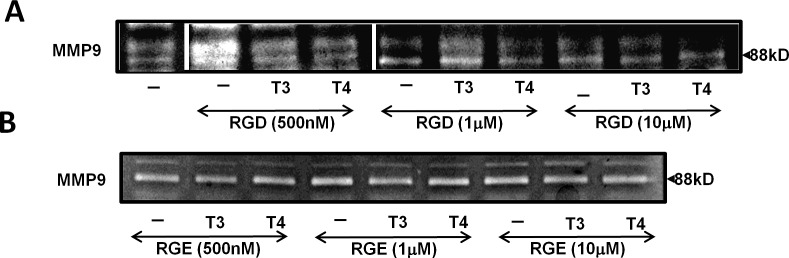
Thyroid hormone-induced MMP-9 activation is blocked by RGD peptide, but not RGE CAG cells (100,000/96-well plate) were incubated under serum-free conditions overnight with (A) RGD or (B) RGE at several concentrations (500 nM, 1 μM and 10 μM) in the presence or absence of T3/T4 (1 nM, 100 nM). Conditioned medium was evaluated for MMP-9 activation by gel zymography. Experiments were repeated twice in duplicate.

After demonstrating that thyroid hormone induces MMP-9 activation via the αvβ3 integrin, we attempted to block this effect. As the hormone-binding site upon the αvβ3 is near the RGD-recognition site and by using RGD peptide the binding of the hormones to the integrin was allosterically blocked [14, 24], we used RGD tri-peptide to hinder the effects of the hormones on MMP-9. CAG cells were treated overnight with T3/T4 (1 nM and 100 nM, respectively) in the presence or absence of several RGD or negative control RGD concentrations (500 nM, 1 μM and 10 μM). Conditioned medium was collected, concentrated and tested for MMP-9 enzymatic activity by gel zymography. MMP-9 activation by T3 was blocked by 25% at the highest concentration of RGD (Figure 3A) and T4-induced MMP-9 activation was significantly reduced by 15-40%, in a dose-dependent manner (p< 0.05). RGD peptide induced MMP-9 activation at nanomolar concentrations. As expected, RGE peptide, used as a negative control in the same experiment (untreated control cells are depicted in Figure 3A, lane 1), did not block thyroid hormones-mediated MMP-9 activation (Figure 3B). Similar results were observed in RPMI-8226 cells (data not shown).

### Tetrac, a selective T3/T4−αvβ3 antagonist, blocks MMP-9 expression and activation

**Figure 4:**
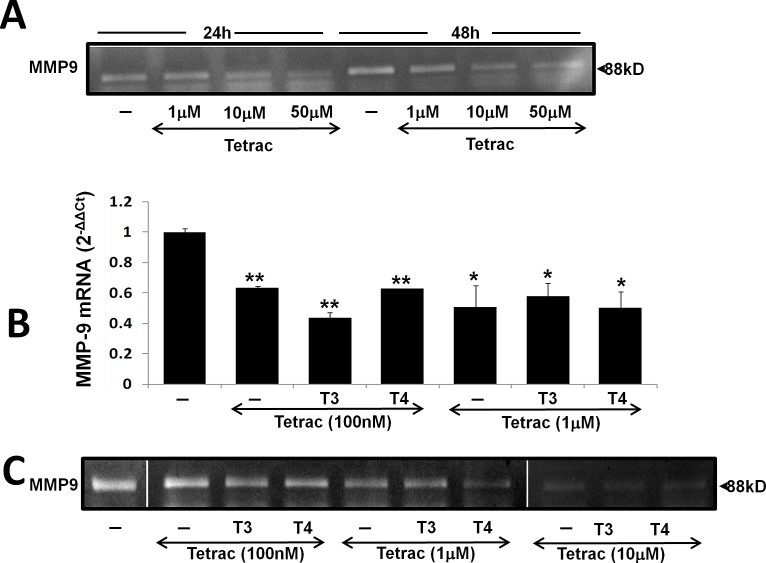
Tetrac blocks thyroid hormone-induced MMP-9 expression and activation CAG cells (100,000/96-well plate) were incubated in serum free conditions (A) for 24-48 h with tetrac (1-50 μM) or for 24h in the presence of tetrac (100 nM-10 μM) with/without T3 (1 nM) or T4 (100 nM) and evaluated for (B) MMP-9 mRNA by real-time PCR expressed as fold-change (2^−ΔΔCt^) relative to control cells and by (C) Gel zymography. Experiments were repeated twice in duplicate. *p<0.05, **p<0.005.

Tetrac was previously shown to block the thyroid hormones-αvβ3 axis in various tumor models including myeloma[15]. We were therefore interested in examining the effect of tetrac on MMP-9 activity and whether it can block thyroid hormones-induced MMP-9 activation. CAG cells were treated with increasing tetrac concentrations (1 μM, 10 μM and 50 μM) for 24-48 h and gel zymography was performed on the conditioned medium. Results indicate that tetrac effectively blocked MMP-9 activity in a dose- and time-dependent manner (Figure 4A). Next, CAG cells were treated with tetrac (100 nM and 1 μM) with/without T3 (1 nM) or T4 (100 nM) and RNA was extracted after an overnight incubation. Real-time PCR results revealed that, at both 100 nM and 1 μg, tetrac alone or in the presence of agonist thyroid hormones (T4 or T3), inhibited MMP-9 mRNA expression (Figure 4B). The inhibitory effect of tetrac on MMP-9 was further examined by gel zymography in CAG cells treated overnight with tetrac (100 nM, 1 μM and 10μM) in the presence of T3 (1 nM) or T4 (100 nM). Conditioned medium samples were loaded in two parallel gels which were run and developed together. Results (Figure 4C), from representative lanes (skipping lanes are clearly marked by separating lines), demonstrate that, in a dose-dependent manner, tetrac effectively blocked MMP-9 activation by the hormones (Figure 4C). Similar results were obtained in RPMI-8226 cells (data not shown). In a parallel set of experiments, the inhibitory effect of tetrac on MMP-9 activity was observed under serum-containing conditions in various myeloma cell lines (Supplementary Figure 1).

### The effect of tetrac and tetrac nanoparticle on MMP-9 activation in primary cells from myeloma patients

**Figure 5:**
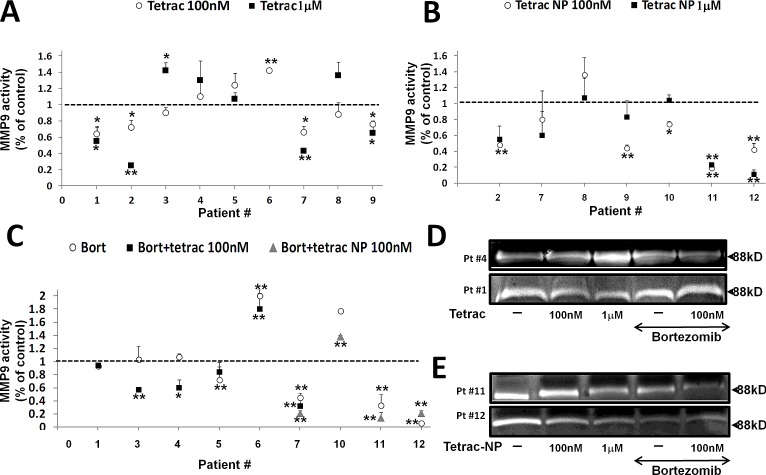
The effect of tetrac and tetrac-NP on MMP-9 activity in primary cells from myeloma BM Mononuclear cells (MNC) from 12 MM patients BM's were collected, seeded (100,000/96-well plate) and incubated for 72-96 h with 100 nM and 1 μM of (A) tetrac or (B) tetrac-NP (C) or combined with bortezomib (25 nM) and evaluated for MMP-9 activation by gel zymography. (D-E) Representative gels from selected patients. Experiments were quantified twice and presented as -fold of control (average ± SD). *p<0.05, **p<0.005.

Next, we examined the effects of tetrac and tetrac-NP on MMP-9 activity in primary cells from myeloma patients. Bone marrow (BM) aspirates were obtained from 12 MM patients, 11 newly diagnosed and 1 at disease relapse (supplementary Table 1). Mononuclear cells (MNC) were separated by Ficoll-Paque and the percentage of plasma cells (CD-138 positive) was determined by flow cytometry (supplementary Table 1) and ranged from 15-96% (average 56 ± 27). Cells were treated with 100 nM and 1 μM tetrac and tetrac-NP for 72-96 h and conditioned medium was collected, concentrated and examined by gel zymography. MMP-9 activity in each sample was calculated as percentage from untreated cells (assigned a value of 1 by definition, marked by a dashed line in Figure 5). Tetrac (Figure 5A) reduced MMP-9 activity below the control level in 6 of 9 examined samples, while tetrac-NP (Figure 5B) inhibited MMP-9 activity in 6 of 7 samples. No effect on MMP-9 activity and protein level was shown by both agents in control primary MNC (Supplementary Figure 2).

### The effect of bortezomib alone or in combination with tetrac/tetrac-NP on MMP-9 activity in primary cells from myeloma patients

Lastly, we evaluated the effect of bortezomib on MMP-9 activity as a single agent or adjunct to tetrac/tetrac-NP. MNC from the BM of nine patients were treated with bortezomib (25 nM) for 72-96 hours under serum-containing media. A reduction in MMP-9 activity by bortezomib was observed in four samples (Figure 5C, white circles). Next, six of the samples were co-treated with bortezomib and 100 nM tetrac (Figure 5C, black squares) and four with tetrac-NP (Figure 5C, grey triangles). For both tetrac and tetrac-NP, results from different BM samples were heterogeneous, with an additive effect in some samples while antagonism in others. In details, in four samples (BM's #3/4/6/7) MMP-9 activity was reduced in the presence of the combined treatment, while no effect (BM#1) or an antagonistic effect (BM#5) was documented. Tetrac-NP, when combined with bortezomib, further inhibited MMP-9 activity in three samples (BM's #7/10/11), while in the fourth sample (BM#12) an antagonistic effect was shown. Representative zymography results from cells treated with tetrac with/without bortezomib showing synergism (Figure 5D, upper panel) or antagonism (Figure 5D, lower panel) are presented. Similar representative results are shown for tetrac-NP with/without bortezomib showing synergism (Figure 5E, upper panel) or antagonism (Figure 5E, lower panel).

## DISCUSSION

In our myeloma experimental model, thyroid hormone increased adhesion to fibronectin and RGD, and enhanced cell migration in a wound healing assay. The regulation of adhesion and migration by T3 [18-21] and by T4 [25], was previously reported in normal physiological processes. However, data regarding their involvement in cancer cell adhesion is limited [22, 23] with no reports in multiple myeloma. The interaction of myeloma plasma cells with fibronectin was reported to be via engagement with αvβ3 integrin [12] and proposed as a novel mechanism for their invasion and spreading. This integrin was recently shown, by our group, to be upregulated in MM cells treated with T3 and T4 [15]. In accord with these observations, we now show that the increased adhesion to fibronectin and RGD by the hormones coincides with αvβ3 clustering upon the MM plasma cells.

A role for the αvβ3 integrin in the production of MMP-9 in myeloma has been reported [9, 12, 26]. This enzyme has a central role in myeloma adhesion, motility and invasion [5, 7, 12, 27-29]. In recent years thyroid hormones involvement in MMP regulation was recognized in normal tissues[18, 30, 31], amphibian development [32] and cancer [33, 34], but not in myeloma. We therefore investigated the effects of thyroid hormones on MMP-9 production in this disease and found that thyroid hormones enhance MMP-9 transcription, protein secretion and enzymatic activity via the αvβ3 integrin. This MMP-9 activation was dependent on ERK phorphorylation, as in previous reports [5, 29, 35]. Our current work demonstrates a novel effect by thyroid hormone via the αvβ3-MAPK axis, a pathway which has been shown to mediate the mitogenic effects of the hormones in myeloma [15].

Given the relevance of MMPs to cancer progression, several MMP inhibitors are under clinical studies [36]. The proteasome is a major cellular protease complex that controls the concentration and turnover of molecules in ECMs, including MMPs [37]. The down-regulation of MMP-9 protein levels by bortezomib, the first proteasome inhibitor in the clinic, has been documented in lung cancer [38], squamous cell carcinoma [39], bladder carcinoma [40], breast cancer cells [41] and myeloma cells [42]. The inhibition of MMP-9 by bortezomib may be due partly to blocking of the activation and nuclear translocation of NF-κB, which was reported to regulate MMP-9 transcription [43]. We present for the first time in myeloma cells that the inhibition by bortezomib of the secreted and activated form of MMP-9, an effect that is antagonized by T3 and T4.

Overall, our novel findings suggest that thyroid hormones might have pleiotropic effects in the process of MM cell trafficking and homing. Therefore, disruption of the thyroid hormones-integrin-MMP-9 signaling may be of importance in myeloma treatment. The effects of two αvβ3 blockers, RGD tri-peptide as well as the specific antagonist tetrac, were studied in myeloma. RGD exhibited a differential effect on thyroid hormone-induced MMP-9 activation. This sub-specialization was previously reported with regard to MAPK and viability [15] and may be due to the existence of two distinctive binding domains for T3 and T4 at the receptor for thyroid hormone on αvβ3 integrin [24]. In our experimental assay RGD, at low nanomolar concentrations, greatly induced MMP-9 activity. This agonistic activity by RGD coincides with previous report indicating stimulation of tumor growth and angiogenesis at low nanomolar concantrations[44]. Tetrac effectively disrupted MMP-9 induction and release by both hormones. This agent has been shown previously to inhibit additional MMP family members (MMP-15 and MMP-19) and induce tissue inhibitor of metalloproteinase (TIMP) in an angiogenic model [45]. MMP inhibitory activity was also shown by resveratrol [46], for which there is a specific receptor proximal to the thyroid hormone/tetrac binding site on αvβ3 [47-49].

In an attempt to demonstrate direct effects on malignant cells with relevant impact on their relationship with the surrounding milieu, tetrac and its nanoparticulate formulation (tetrac-NP) were also examined in mononuclear cells from MM bone marrow samples. Our results indicate that tetrac and tetrac-NP, at low molar concentrations, effectively blocked MMP-9 production in the majority of samples examined while no effect was observed in a control BM sample. When we examined whether tetrac can be combined with bortezomib, results from different BM samples were heterogeneous, with an additive effect in some samples and antagonism in others. Whether tetrac could be combined with bortezomib or other anti-myeloma drugs, is under active investigation.

To conclude, we have examined the role of thyroid hormones on multiple myeloma cell adhesion, migration and MMP-9 secretion. We demonstrated that thyroid hormones promote the adhesion of myeloma cells and that this interaction triggers clustering of αvβ3 integrin and the biosynthesis of the matrix degrading enzyme, MMP–9. The thyroid hormone-dependent regulation of MMP-9-integrin interactions defines a novel mechanism that may play a role in myeloma cell migration and disease progression.

## METHODS

### Cell lines and primary cells

MM cell lines CAG (established at the Arkansas Cancer Research Center from bone marrow aspirates of patients with MM) and RPMI-8226, U266 (CCL 155 and TIB-196, respectively, ATCC; Rockville, MD, USA) were maintained in RPMI 1640 medium supplemented with 10% heat-inactivated FBS/antibiotics. Bone marrow (BM) aspirates were obtained upon written consent from 12 MM patients (Supplementary Table 1) treated at the Meir Medical Center (51-89 years old). The Meir Medical Center Helsinki Committee approved this study (#0205-12-MMC). 11 samples were taken at diagnosis and one during disease relapse. Another sample served as control and was taken from a patient with an infectious disease and without any bone marrow involvement. The BM mononuclear cells (MNC) were isolated by Ficoll-Paque gradient centrifugation per the manufacturer's instructions (Sigma-Aldrich, St. Louis, MO, USA). Percentage of plasma cells (CD-138+) and their isotype was evaluated by flow cytometry. For experiments, the cells were seeded in 96-well plates (100,000 cells/well) in serum-containing/ free RPMI 1640 medium and treated with T3 (1 nM), T4 (100 nM), tetrac or tetrac-NP (100 nM-100 μM) with or without bortezomib (25 nM) for 24-96 h.

### Reagents and chemicals

T3, T4 and tetrac (Sigma-Aldrich, St. Louis, MO, USA) were dissolved to 1 mM in KOH-PG (final concentration of 0.04 N KOH with 0.4% polyethylene glycol (vol/vol). RGD/RGE peptides (Sigma) were dissolved to 100mM in PBS). Bortezomib was obtained from the oncology pharmacy at Meir Medical Center. Vehicle control was used in each experiment. APC-CD138 antibodies (clone B-B4) were from Miltenyi Biotec, Bergisch Gladbach, Germany. αvβ3 (LM609 unconjugated/PE) monoclonal antibody was from Chemicon International, Harrow, UK. U0126 was purchased from Cell Signaling Technology, Danvers, MA, USA and MMP-9 antibody (#3852) was obtained from Cell Signaling (Boston, MA, USA).

### Adhesion assay

For adhesion assays 96-well plates were pre-coated for 30 minutes with fibronectin (15 μg/mL), RGD (2.5 μg/mL) or BSA (10 μg/mL), washed twice with PBS and blocked for 30 minutes in BSA (2mg/mL). CAG cells were seeded (100,000 cells/24-well plate) under serum-free conditions for 24 hours before the addition of T3 (1 nM) or T4 (100 nM) for an overnight incubation. Afterwards cells were collected, counted and an equal number of cells were re-seeded for 30 minutes in 37º C in the pre-coated plates (50,000/96-well plate). Unattached cells were washed twice with PBS and adhered cells were stained with Hoechst 33342. Cells were visualized by a florescent microscopy equipped with a camera (Model IX71, Olympus, Hamburg, Germany) with a 20X/0.50 objective lens and Cell^A (Version 3.1) Olympus software imaging.

### Immunofluorescence

Treated cells were fixed and permeabilized with 0.1% Triton x-100 for 5 min at room temperature and then incubated with Alexa Fluor 488- labeled Phalloidin (A12379, Molecular Probs, Inc, OR, USA) and PE-conjugated αvβ3 antibody (LM609, Chemicon International). Hoechst 33342 was used for nuclear staining (Sigma-Aldrich). Cells were visualized by a fluorescent microscopy equipped with a camera (Model IX71, Olympus, Hamburg, Germany) with a 20X/0.50 objective lens and Cell^A (Version 3.1) Olympus software imaging.

### Wound healing assay

CAG cells were seeded (100,000 cells/96-well plate) and starved for 48 h in serum-free media. Each well was scratched in the middle and T3 (1 nM) or T4 (100 nM) were added for 0, 24, 48, 72 and 96 h. Cells were visualized by light and florescent microscopy equipped with a camera (Model IX71, Olympus, Hamburg, Germany) with a 20X/0.50 objective lens and Cell^A (Version 3.1) Olympus software imaging at each time point. After 96 h, cells were fixed with 4% paraformaldehyde and permeabilized with 0.2% Triton x-100 for 5 min. 1% BSA was used for blocking, followed by Hoechst and Phalloidin staining.

### RNA extraction and cDNA synthesis

RNA was extracted using NucleoSpine RNA II kit (Macherey-Nagel, Düren, Germany) according to the manufacturer's instructions and eluted in 40 μL RNase free water. RNA concentration and purity were measured using NanoDrop™ 1000 Spectrophotometer (Thermo Scientific, Wilmington, DE, USA). RNA (200 ng) was reverse-transcribed using High Capacity cDNA Reverse Transcription Kit (Applied Biosystems, Carlsbad, CA, USA), according to manufacturer instructions.

### Real-Time PCR

MMP-9 mRNA levels were measured by Real-Time PCR (7500 Fast system, Applied Biosystems, Carlsbad, CA, USA), using Fast Sybr Green Mix (Applied Biosystems). Results, normalized to *actin beta*, were calculated as fold change using the comparative threshold cycle method (2^−ΔΔCT^) relative to control cells (i.e., controls are assigned a value of 1 by definition). Primers (Hylabs, Israel) were designed (Primer-Express software, Applied-Biosystems) in different exons in order to elimi­nate DNA contamination.

*MMP*-9 forward primer: GCCACTACTGTGCCTTTGAGTC and *MMP*-9 reverse primer: CCCTCAGAGAATCGCCAGTACT.

*Actin beta* forward primer: CCTGGCACCCAGCACAAT and *actin beta* reverse primer: GCCGATCCACACGGAGTACT.

### Western blotting

Equal numbers of MM cells were seeded and treated, then equal volumes of conditioned medium from each experiment were collected and 12-fold concentrated by centrifugal filter devices for proteins with molecular weight over 30kD (Amicon Ultra, Millipore). Half of the concentrated supernatant (20 μL) was separated on 10% polyacrylamide gels and analyzed by western blot, using above indicated primary antibodies and appropriate secondary HRP-conjugated antibody (Jackson ImmunoResearch Laboratories, West Grove, PA, USA). Immunoreactive proteins were detected by chemiluminescence reagents (Beit-Ha'emek, Israel). Ponceau (Sigma Aldrich) was used for quantitation to normalize the proteins loaded onto the membrane. Band intensity was visualized and quantified using LAS-3000 (FujiFilm, Japan).

### Gelatin Zymography

The activity of MMP-9 was analyzed by gelatin zymography. Equal numbers of MM cells in sub-confluent culture conditions (about 70 – 80%) were incubated with the different treatments for the indicated times. Equal volumes of supernatant (500 μL) were collected and concentrated 12-fold by centrifugal filter devices for proteins with molecular weight over 30kD (Amicon Ultra, Millipore). Half of the concentrated supernatant (20 μL) was taken for gel zymography and were electrophoresed under non-reducing conditions on 10% SDS-polyacrylamide gels impregnated with 1 mg/mL of gelatin before casting. Gels were washed for 30 min in 2.5% Triton X-100 to remove the SDS, then incubated overnight at 37°C in digestion buffer (50 mM Tris-HCl and 10 mM CaCl2, pH 7.4). Gels were stained with Coomassie Blue (R250) for 30 min and destained with methanol:acetic acid:water (50:10:40). Areas of protease activity appeared as clear bands on a dark blue background. Band intensity was visualized and quantified using LAS-3000 (FujiFilm, Japan) and normalized to for 18 h at 37°C.

### Statistical analysis

Experiments were analyzed using Student's unpaired t-test for significance (p< 0.05) and results are presented as average±SD.

## SUPPLEMENTARY INFORMATION TABLE AND FIGURES


