# Supplementary material for: Thyroid hormone regulates adhesion, migration and matrix metalloproteinase 9 activity via αvβ3 integrin in myeloma cells
Source: Oncotarget. 2014 Jul 13;5(15):6312–22. doi: 10.18632/oncotarget.2205 (PMC4171632; doi:10.18632/oncotarget.2205)
Supplement: Supplementary file 1 [file oncotarget-05-6312-s001.pdf]

Thyroid hormone regulates adhesion, migration and matrix metalloproteinase 9 activity via αvβ3 integrin in myeloma cells

Supplementary Information

Supplementary Table 1: Patients data

Multiple myeloma patients				
**Pt #**	**Age/Sex**	**Stage**	**Isotype**	**% CD138+ / MNC's**
1	60 M	Diagnosis	IgG k	96%
2	59 M	Diagnosis	FLCk	83%
3	82 M	Diagnosis	IgG k	42%
4	72 M	Diagnosis	IgA k	82%
5	68 M	Diagnosis	IgG k	37%
6	60 F	Diagnosis	IgG λ	82%
7	89 M	Diagnosis	IgA k	15%
8	54 F	Diagnosis	IgG k	41%
9	68 F	Relapsed	IgG λ	39%
10	51 F	Diagnosis	IgG λ	62%
11	56 F	Diagnosis	IgG k	24%
12	56 F	Diagnosis	IgA λ	73%
13	56 M	Control	N/A	< 5%

Pt#, patient number; M, Male; F, Female; Age (years); MNC, Mononuclear cells

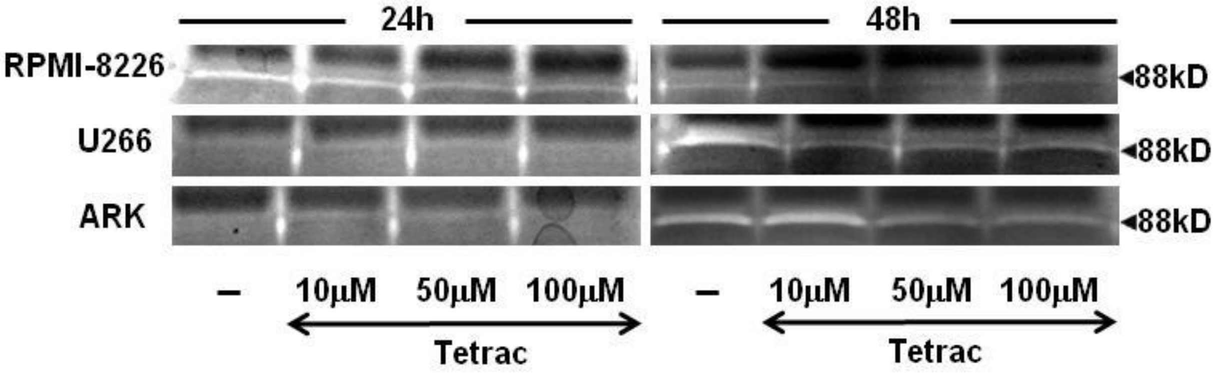

Supplementary Figure 1: Tetrac blocks MMP-9 activation in several MM cell lines. RPMI-8226, U266 and ARK cells (100,000/96-well plate) were incubated for 24-48 h with tetrac (10-100 μM) and evaluated for MMP-9 activity by gel zymography. Experiments were repeated at least twice in duplicate.

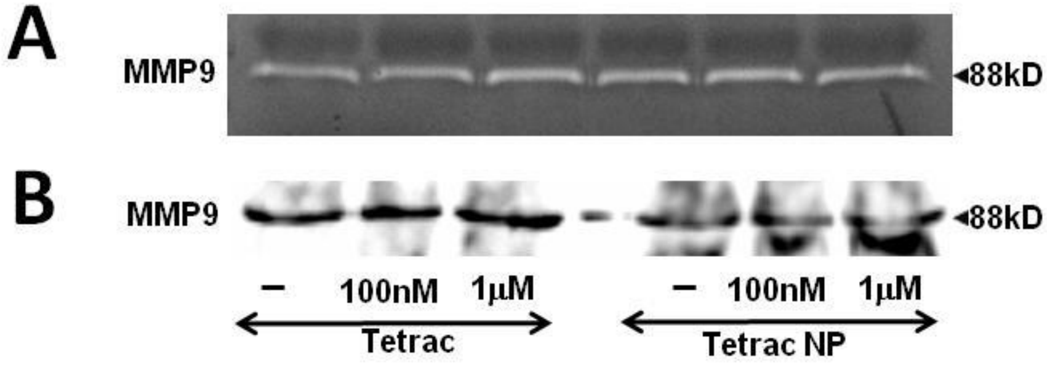

Supplementary Figure 2: No effect on MMP-9 activation of tetrac in control cells. MNC from pt.#13 (100,000/96-well plate) were incubated for 96 hours with tetrac or tetrac-NP (100 nM and 1 μM) and evaluated for (A) MMP-9 activity by gel zymography and (B) MMP-9 protein level by western blot.
